# Network Analytics Enabled by Generating a Pool of Network Variants from Noisy Data

**DOI:** 10.3390/e25081118

**Published:** 2023-07-26

**Authors:** Aamir Mandviwalla, Amr Elsisy, Muhammad Saad Atique, Konstantin Kuzmin, Chris Gaiteri, Boleslaw K. Szymanski

**Affiliations:** 1 Department of Computer Science, Rensselaer Polytechnic Institute, Troy, NY 12180, USA; mandva@rpi.edu (A.M.); amrelsisy@ymail.com (A.E.); msaadatiq@gmail.com (M.S.A.); kuzmik2@rpi.edu (K.K.); 2 Network Science and Technology Center, Rensselaer Polytechnic Institute, Troy, NY 12180, USA; 3 Rush Alzheimer’s Disease Center, Rush University Medical Center, Chicago, IL 60612, USA; christopher_gaiteri@rush.edu; 4 Department of Psychiatry, SUNY Upstate Medical University, Syracuse, NY 13210, USA

**Keywords:** functional and structural uncertainty, noisy data, covert networks, Bernoulli weighted random network generator

## Abstract

Mapping network nodes and edges to communities and network functions is crucial to gaining a higher level of understanding of the network structure and functions. Such mappings are particularly challenging to design for covert social networks, which intentionally hide their structure and functions to protect important members from attacks or arrests. Here, we focus on correctly inferring the structures and functions of such networks, but our methodology can be broadly applied. Without the ground truth, knowledge about the allocation of nodes to communities and network functions, no single network based on the noisy data can represent all plausible communities and functions of the true underlying network. To address this limitation, we apply a generative model that randomly distorts the original network based on the noisy data, generating a pool of statistically equivalent networks. Each unique generated network is recorded, while each duplicate of the already recorded network just increases the repetition count of that network. We treat each such network as a variant of the ground truth with the probability of arising in the real world approximated by the ratio of the count of this network’s duplicates plus one to the total number of all generated networks. Communities of variants with frequently occurring duplicates contain persistent patterns shared by their structures. Using Shannon entropy, we can find a variant that minimizes the uncertainty for operations planned on the network. Repeatedly generating new pools of networks from the best network of the previous step for several steps lowers the entropy of the best new variant. If the entropy is too high, the network operators can identify nodes, the monitoring of which can achieve the most significant reduction in entropy. Finally, we also present a heuristic for constructing a new variant, which is not randomly generated but has the lowest expected cost of operating on the distorted mappings of network nodes to communities and functions caused by noisy data.

## 1. Introduction

The amount of data collected in the world has grown exponentially for at least the last decade [1], including data on covert networks [2]. To capitalize on such network data, access to it needs to be supplemented with tools capable of curating data and extracting key results. Specifically, the analysis of real-world networks needs to overcome errors recorded in the network data that occur during the acquisition process. For small datasets it may be possible to correct these errors manually, but it is not feasible for large datasets, especially when edges are purposefully added or disguised by actors in the network. Therefore, we propose a new method to deal with networks created from data with unintentional or intentional errors, which can significantly improve downstream extraction of key network features.

The sources of noise in network data can be classified into several categories. The first source of noise originates from monitoring relations that are not directly observable, so collected data are only a proxy of the desired relationships. In the context of social networks, an example of this would be frequent communication between two people which may imply a trust relationship between them. To illustrate the potential shortcomings of such proxies, we can observe that some of the calls might be strictly professional or even an indication of disagreement and distrust rather than trust. Similarly, in a biological network context, we rely on proxies for protein interactions (their physical binding) in artificial testing systems. These proxies can produce many false positives, as bound proteins might never actually be found in the same place or at the same time within their originating cell [3].

The second category of noisy data is caused by deliberate distortion or attempts to conceal some characteristics of the network, or even its entire existence, undertaken by the nodes of such a network. The best example of this category of noise would be covert networks, where members of the network are intentionally hiding their involvement and interactions by avoiding communicating within the network in cases when the only available means of communication can be easily tracked, such as cell phones with registered ownership [4,5]. To avoid detection of their interactions within crime organizations, the criminals may use wiretapped phones only for private conversations [6]. Finally, in many networks with massive data collections, the third category of sources of data noise is the presence of a low but persistent rate of erroneous experimental measurements that distort the valid results [7,8].

The presence of noise in network data distorts the detection of network edges, which is likely to modify the network’s community structure, e.g., [9]. In the absence of the ground-truth data about basic properties of the network, like the allocation of nodes to communities and to network functions, an operation designed based on a single network derived from such data will not be able to predict all distortions that can arise during such an operation.

The rest of this paper is organized as follows. Section 2 contains a brief review of the relevant literature. Section 3 describes the methodology used in our paper. In Section 4, we present the datasets used for the experimental evaluation of the results. The design of the experiments and the results are presented in Section 5. The summary of the work, the significance of our findings, and some concluding remarks are given in Section 6.

## 2. Related Work

To address the problem of missing, mislabeled, or incorrectly included nodes or edges in a network, several approaches have been proposed. Many of them use entropy-based metrics. In [10], the authors measure the uncertainty of nodes considered for strengthening or weakening of their existing links with the neighbors. In [11], an entropy-based metric is used to measure the vulnerability of communities in complex networks to breaking up. Similar metrics are also used to measure the structural similarity of nodes in complex networks based on the local structure topology of each node [12]. In [13], the authors present a successful example of applying entropy metrics to measure the evolution of human communications. The authors found that Shannon’s entropy tends to decay over time when the social network stagnates due to experiencing little or no changes among members that comprise the network.

In [14], the authors consider the temporal evolution of networks and observe the network structure at each time step in conjunction with prior distributions from the history of network changes. According to the authors, their method leads to more robust communities that are less influenced by noisy collected data and results in networks that are less likely to undergo dramatic changes over a short time span.

In [15], the problem of noisy networks is approached by pruning less prominent edges to create a backbone of a network. The backboning method used by Coscia et al. [15] views nodes not just as a source of edges but considers the nodes’ ability to send and receive communications. It evaluates the significance of each edge and tags weak connections that do not translate into significant interaction between nodes as noise. The noise-corrected approach described by the authors is scalable and correctly estimates the variance of the transformed edge weights while ensuring high quality of the backbones as shown by a series of Ordinary Least Squares (OLS) regressions.

## 3. Methods

In our earlier work [16], we focused only on the cost of assigning a node to the wrong community. In this section, we introduce preprocessing steps that are applied to the collected noisy network data, three novel entropy-based metrics, and two new heuristics, each of which constructs the community structure for a given network while minimizing the expected cost arising from operating on networks with communities and functions distorted by using noisy data for their creation.

Using the Bernoulli weighted random network (BWRN) algorithm, we generate a set of *r* networks from the given noisy network data and find their non-overlapping communities. Then, we cluster these networks into s≤r groups of networks that share the same community structure. Because the network community structures are robust to minor edge perturbation, for large *r* and s<r, the ratio of the size of each cluster to *r* approximates the probability that the corresponding structure is the ground truth. In this work, to quantify the uncertainty of a community structure and its corresponding level of predictability, we introduce three entropy-based metrics that are adopted from human mobility entropy models proposed in [17].

As discussed in Section 2, given the unavailability of the ground truth, using only a single network derived from noisy data is incapable of predicting all distortions in the underlying data. To address this weakness, we apply a generator that rewires networks using a combination of the Stochastic Block Model (SBM) [18] and hierarchical model [19]. The SBM is useful due to its ability to limit the changes to the community structure of the generated network, while a hierarchical model helps preserve the network’s member hierarchy. There are other generative models that can be used to create networks with communities, some of which are discussed in [20]. The extent of rewiring is controlled by a user-provided parameter $p_{B}\in \left(0,1\right]$, which defines the variance of the generated weights distribution. As $p_{B}$ approaches 1, the rewired networks become more like each other, and the original noisy network [16].

Given the basic noisy parameters of such a network, including the lists of nodes, weighted degrees of all nodes, communities, and hierarchy, the generator produces a set of randomly generated networks by randomly redirecting weak edges while preserving the strong ones. In the process of generating a vast number of statistically equivalent networks, the generator records the network structure for each unique network variant. Any duplicates of an already recorded network just increase the duplicate count for this network.

When all networks are generated, we treat each unique network as a solution variant and assign to it the probability that this variant represents the ground truth. This probability is approximated by the ratio of the count of this network’s duplicates plus one to the total number of all generated networks. Variants with large occurrence counts correspond to networks with the most persistent patterns of network structures. Using just the Shannon entropy, we can select a variant that minimizes the uncertainty for operations planned on the network. Repeatedly generating new pools of networks from the resultant variant lowers the entropy of the result. Moreover, if the entropy or the cost of distortions is too high, the network operators can identify nodes, monitored for which can fastest reduce the entropy.

We apply the entropy-based metrics described in Section 2 to a set of *s* community structures derived from *r* networks generated by the BWRN algorithm [16]. In our application, entropy-based metrics are used to measure the uncertainty arising from two probabilities assigned to each node. The first is being a member of a community, referred to as structural uncertainty, and the second is performing the function assigned to this node, referred to as functional uncertainty. These uncertainties are measured in the set of generated community structures and functions assigned to nodes. The goal is to construct a community structure with the lowest expected cost of operating on a network with uncertain communities and node functions caused by noisy network data. We present two new heuristics to create such community structures. They are important in the investigation of criminal or terrorist organizations and in planning their disruptions.

### 3.1. Preprocessing of Noisy Network Data and Shannon Entropy Metric

Given a network with a set *N* of nodes, denoted as $\left\{n_{1},n_{2},\ldots ,n_{\left|N\right|}\right\}$, and a set E⊆N×N of edges, we use the BWRN generator [16] to rewire the given network *r* times, creating a set of *r* networks which are statistically equivalent to each other. Then, we use the Louvain community detection algorithm [21] to detect non-overlapping communities in each generated network. We cluster these networks into s≤r groups, each containing the same community structure $C_{i}$ for i=1,…,s. Each community structure $C_{i}$ has weight $w_{i}$ defined as the number of networks that share this structure.

This set of *s* community structures is subsequently used as a proxy for the ground-truth community structure for the given noisy network data. When r→∞, fractions $f_{i}^{C}=w_{i}/r$ asymptotically converge to the probability that the community structure $C_{i}$ is the ground truth for the given noisy network data.

The preprocessing steps expect that the values for the following parameters are selected: $p_{B}$, which controls the extent of rewiring, and *r*, which defines the number of generated networks. The smaller $p_{B}$ is, the larger *r* must be, because more aggressive rewiring requires generating more networks to create all feasible community structures. Because the methods presented here are heuristics, they require finding suitable values of these parameters to obtain the best results.

The first entropy-based metric proposed here is the classic Shannon entropy computed over an entire set of generated community structures by setting $p_{i}^{C}=f_{i}^{C}$. The corresponding equation is

(1) $$e_{s}^{C}=\sum_{i=1}^{s}p_{i}^{C}\ln p_{i}^{C}$$

It follows from the definition that the most reliable ground-truth structure is the community structure $C_{i}$ with the highest fraction of $f_{i}^{C}$.

### 3.2. Set Entropy-Based Metrics

In this section, we introduce three entropy-based metrics that adopt the human mobility entropy metrics proposed in [17] for use in our application. This reference uses mobile phones and tracks users’ locations by identifying cell towers servicing the call of each user as the location. The authors define uncertainty of user location by introducing three entropy metrics for increasingly complex mobility patterns of the cell towers servicing the calls. The first is the random entropy defined as

(2) $$S_{i}^{rnd}=log_{2}V_{i},$$

where $V_{i}$ is the number of distinct locations (cell towers) visited by user *i*. The second is the temporal-uncorrelated entropy, defined as

(3) $$S_{i}^{unc}=-\sum_{j=1}^{V_{i}}p_{i}\left(j\right)\;log_{2}p_{i}\left(j\right),$$

where $p_{i}\left(j\right)$ is the historical probability that location (cell tower) *j* was visited by user *i*. The third and final measure is the real entropy $S_{i}$, which depends on the frequency and order of visits made by each user. Let $T_{i}=X_{1},X_{2},\ldots ,X_{L}$ denote the sequence of cell towers at which that user *i* was observed at each consecutive hourly interval. Then, the real entropy is

(4) $$S_{i}=-\sum_{T_{i}^{\prime }\subset T_{i}}P\left(T_{i}^{\prime }\right)\;log_{2}\left[P\left(T_{i}^{\prime }\right)\right],$$

where $P(T_{i}^{\prime })$ is the probability of finding a particular time-ordered subsequence $T_{i}^{\prime }$ in the trajectory $T_{i}$. The authors also introduce important measures of predictability $\Pi \leq \Pi^{max}\left(S,V\right)$, where $\Pi^{max}$ represents the maximum predictability for each user, and is calculated as

(5) $$S=H\left(\Pi^{max}\right)+\left(1-\Pi^{max}\right)log_{2}\left(V-1\right),$$

where the binary entropy function is

(6) $$H\left(\Pi^{max}\right)=-\Pi^{max}log_{2}\left(\Pi^{max}\right)-\left(1-\Pi^{max}\right)log_{2}\left(1-\Pi^{max}\right).$$

The maximum predictability for $\Pi^{rnd}$ and $\Pi^{unc}$ is also determined and extracted from $S^{rnd}$ and $S^{unc}$, respectively. For the real entropy, *S*, we map each node onto the mobile user. Time slot *t* in the mobility model is mapped to community structure $C_{t}$, where the node $n_{i}$ visits all cell towers associated with members of its community in $C_{t}$, during time slot *t*. Finally, we impose the order of visitations from the most to least frequent pairings between the node $n_{i}$ and each member of its communities. In other words, node $n_{i}$ will first visit its community member $n_{j}$ that most frequently appears with $n_{i}$ in the same communities across all *s* community structures.

### 3.3. Selecting the Community with the Smallest Expected Cost of Structural and Functional Uncertainties

So far, we have used the entropy measures to find the community structure $C^{can}$ with the highest approximated probability to be the ground-truth structure and therefore having the lowest entropy. Here, we select a new structure that ensures the lowest expected cost of operating on a network with uncertain communities and node functions. This cost, $C_{T}$, is defined by a pairwise comparison of the newly constructed *k* version of a candidate community structure $C_{k}^{can}$ to each of the *s* already established structures $C_{i}$. This cost can be defined as

(7) $$C_{T}\left(C_{k}^{can}\right)=\sum_{i=1}^{s}C\left(C_{k}^{can},C_{i}\right)$$

To demonstrate how to construct such cost functions, we introduce two simple but useful examples of them using pairs of community structures $C_{k}^{can},C_{i}$, shown in Equation (7). We call the first cost function frequency based as it accounts for the average frequency of pairs of nodes appearing in all ground-truth communities. It is defined as follows:

(8) $$C_{freq}\left(C_{k}^{can},C_{i}\right)=\sum_{j=1}^{\left|N\right|}\left(|c_{k,j}^{can}\cup c_{i,j}|-|c_{k,j}^{can}\cap c_{i,j}|\right)f_{i}^{C}.$$

where $c_{k,j}^{can}$ denotes the community with node $n_{j}$ in $C_{k}^{can}$ while $c_{i,j}$ refers to the community with node $n_{j}$ in community structure *i*. Hence, this metric penalizes unmatched members of either community with a unit cost, independent of the community size.

The second cost function, named the fraction-based cost, is defined as

(9) $$C_{frac}\left(C_{k}^{can},C_{i}\right)=\sum_{j=1}^{\left|N\right|}\left(1-\frac{|c_{k,j}^{can}\cap c_{i,j}|}{|c_{k,j}^{can}\cup c_{i,j}|}\right)f_{i}^{C},$$

Hence, this metric computes an arithmetic complement of the Jaccard similarity metric [22] between pairs of communities that share a node in the corresponding communities $C_{k}^{can},C_{i}$. Unlike the first one, this function discounts the expected cost of unmatched nodes in large communities.

In both cases, the heuristics for creating $C^{can}$ start with the initial $C_{can,1}$ in which each node $n_{j}\in N$ is a community. Let $M_{j}$ denote the average number of members of communities containing node $n_{j}$ in all *s* ground-truth community structures. Then, the total penalty for the initial structure $C_{can,1}$ is $-\left|N\right|+\sum_{j=1}^{\left|N\right|}M_{j}$ for the frequency-based penalty. Denoting $m_{i,j}$ the number of members of a community containing node $n_{j}$ in the $C_{i}$ community structure, the fraction-based penalty can be expressed as $\left|N\right|-\sum_{j=1}^{\left|N\right|}\sum_{i=1}^{s}\frac{f_{i}^{C}}{m_{i,j}}$.

For the frequency-based penalty, we first compute the average frequencies of all pairs of nodes in all *s* feasible ground-truth community structures and denote them as $f_{p}\left(j_{1},j_{2}\right)$. Consequently, the change in penalty from joining $j_{1},j_{2}$ into one community is $p_{c}(1-2f_{p}\left(j_{1},j_{2}\right)$ and the penalty decreases when $f_{p}\left(j_{1},j_{2}\right)>1/2$. This argument holds if we apply it to communities $c_{a},c_{b}$ and consider the frequency of their union $c_{a}\cup c_{b}$. This observation motivates our heuristic, defined inductively as follows:

1. Initial step 1, the initial $C_{can,1}$ is the set of |N| communities, each containing a different single node.
2. Inductive step 1<k≤|N|. Having a community structure with |N|+2−k, we measure the penalty change from merging any pair of communities. Next, we select the pair of communities $i_{1}^{can},i_{2}^{can}$ with the lowest penalty change $p_{c}$ in the merger. If $p_{c}\geq 0$, then the current community structure $C_{can,k-1}$ is the best. Otherwise, we merge communities $i_{1}^{can},i_{2}^{can}$, creating the $C_{k}^{can}$ structure with one less community that is merged with another, which is with |N|+1−k communities. Naturally, this heuristic runs at most |N| steps.

The heuristic for the fraction-based penalty uses the same inductive scheme of merging one pair of communities in each step, selecting a pair whose merging decreases the penalty the most, and stopping when none decreases the penalty.

Both heuristics required careful implementation to be efficient, like creating a dictionary of all *s* community structures, and recomputing frequencies of only a pair of the candidate communities that were merged, which sped up the processing 100 times compared to the initial prototype.

## 4. Data

To present our proposed entropy-based metrics in action, we evaluate them on the real-world Caviar gang [23] and Sicilian mafia [6] criminal networks and the Jakarta Bombing terrorist network [24].

The Caviar network represents criminals who smuggled hashish and cocaine into Montreal, Canada. The data were collected between 1994 and 1996. During this time, the police seized shipments of drugs but delayed any arrests until the investigation was completed. The Caviar network is a weighted and directed network, where edges represent wiretapped telephone calls between members of the network.

The Sicilian network was a drug-trafficking criminal organization based in Sicily, Italy. Its data was collected between 2003 and 2007. This network is also weighted and directed, with edges representing wiretapped telephone calls among members of the network. Both the Caviar and Sicilian networks were derived from data publicly released from court proceedings.

The Jakarta Bombing terrorist network is an undirected weighted network composed of two snapshots showing the network before and after the 2009 Jakarta bombing in Jakarta, Indonesia. We only focus on the pre-attack snapshot because the network was denser before rather than after the attack.

## 5. Results

In the evaluation of our proposed entropy-based metrics and community prediction methods, we use the BWRN generator with $p_{B}=0.875$ to rewire r=1000 networks, using the Caviar and Sicilian criminal networks and the Jakarta Bombing terrorist network. We then measure the Shannon entropy and the set entropy across the community structures of the rewired networks.

### 5.1. Variance of BWRN Generated Networks

The user-defined $p_{B}\in \left(0,1\right]$ controls the variance of the generated weights distribution of the BWRN rewired networks. As $p_{B}$ approaches 1, the rewired networks become more statistically equivalent to the original network. To determine which $p_{B}$ value to use, we test the following values $p_{B}$: [0.5,0.75,0.875,0.9375]. Table 1 shows the resulting Shannon entropy values. As $p_{B}$ increases, the Shannon entropy mean and standard deviation of the community structures generated by BWRN decrease. We find that $p_{B}=0.875$ results in rewired network community structures with the largest range of Shannon entropy values, in comparison to the rest of the tested $p_{B}$ values. Yet, networks rewired using $p_{B}=0.875$ are more statistically equivalent to the original network than networks rewired using smaller $p_{B}$ values, as shown in Table 1. Using $p_{B}=0.9375$, or a larger value, results in the rewired networks and the original network becoming too alike. Therefore, for the rest of this paper, all the BWRN rewired networks will use $p_{B}=0.875$.

### 5.2. Repeated Rewiring of the Network with the Lowest Shannon Entropy

The rewiring of the Caviar and Sicilian networks using the BWRN generator [16] results in creating networks with varying edges and community structures. We use the following heuristic to find a community structure with the lowest Shannon entropy. It starts with rewiring the original network *r* times. In the set of rewired networks, we find the community structure $C^{can}$ with the highest fraction $f_{i}^{C}$ and mark all rewired networks with this community structure as candidates. Using the BWRN generator [16], we rewire every candidate network *r* times and mark the candidate network that results in the set of rewired networks with the lowest Shannon entropy as $g^{can}$. We repeat this process iteratively on the sets of networks rewired using the subsequent $g^{can}$. This process stops when the Shannon entropy of the newly rewired network community structures stops decreasing. Once this happens, we repeat the process one more time, and if all newly rewired networks have a Shannon entropy higher than the previous minimum, we stop. Otherwise, we restart the rewiring to search for the next local minimum of the Shannon entropy, and after finding it, we stop. We found that the Shannon entropy of the networks rewired from the subsequent graph $g^{can}$ tends to be lower than that of the networks rewired from the original network.

We then use the set entropy-based metrics to measure the uncertainty present across the community structures of all the rewired networks. We find that over the first few rounds of rewiring, the Shannon entropy and the set entropy are constantly decreasing, until they reach a minimum value. Once this happens, further rewiring would cause the Shannon entropy and set entropy to increase. What we found to be very interesting is that further rewiring of the resulting networks with an increased entropy value brings back previously observed minimum values of the Shannon entropy and set entropy.

**Figure 1:**
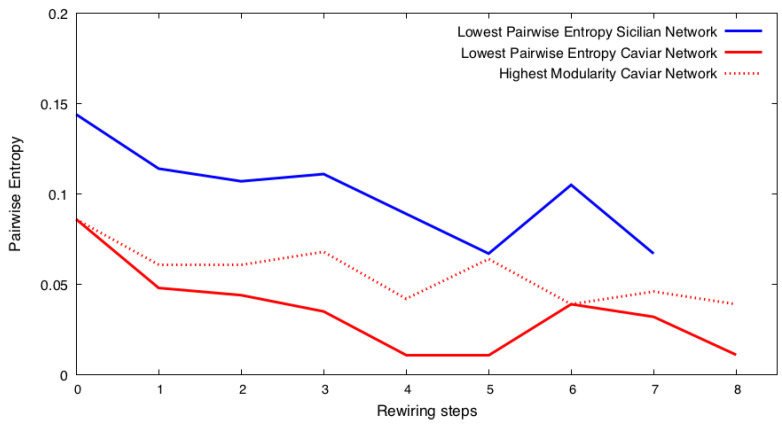
Starting with the rewiring of the original Caviar and Sicilian networks at step 0, we rewire the networks using the BWRN generator [16]. Then, we find a candidate for the lowest Shannon entropy community structure, denoted $C^{can}$, and rewire this structure’s networks to find the one, $g^{can}$, whose rewired networks yield the lowest Shannon entropy. We repeat this process until the minimum Shannon entropy value of the results stops decreasing. The Caviar network creates several candidates $g^{can}$ before getting to the solution. Instead, to speed up the process, we select one candidate network with the largest modularity among all candidates. The figure shows the results of rewiring using the brute-force method versus the heuristic, showing that the latter is more efficient.

The presence of many candidate networks to rewire among the networks rewired from the original network indicates that the original network has low uncertainty, resulting in many networks with the same community structure. Rewiring of O(r) networks *r* times, each with *N* nodes and *L* edges, takes $O(r^{2}gNL)$, where *g* is the cost of generating a random number. For a larger *r*, rewiring all candidates may become infeasible. To continue our process of rewiring efficiently, we use a heuristic that selects a single network with the highest modularity, instead of rewiring. Because the complexity of modularity is O(NL) [25], the heuristic complexity is O(rNL), so it is O(rg) faster than rewiring. As shown in Figure 1, this heuristic works well.

**Figure 2:**
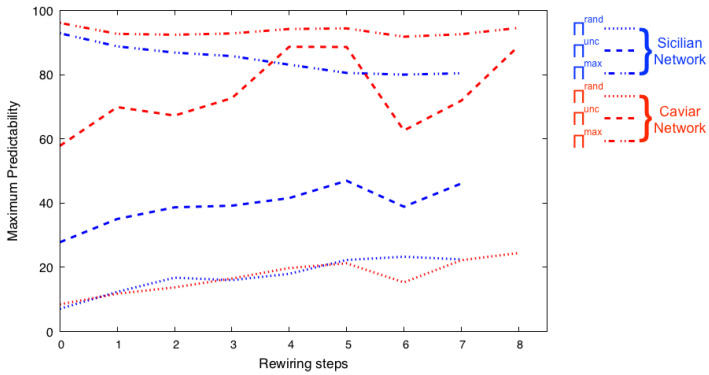
During the iterative rewiring presented in Figure 1, we measure the set entropy and the maximum predictability of the set of rewired network community structures. The three entropy measures used are $S_{i}^{rnd}$, $S_{i}^{unc}$, and $S_{i}$, and we extract from them their corresponding predictability $\Pi^{rnd}$, $\Pi^{unc}$, and $\Pi^{max}$. We find that as the Shannon entropy of $C^{can}$ decreases, so does the set entropy of the best community structure among all rewired networks, signaling the corresponding predictability increases.

In Figure 2, we show the set entropy of the networks generated from the best candidate network $g^{can}$, presented in Figure 1. After every step of rewiring, we measure $S_{i}^{rnd}$, $S_{i}^{unc}$, and $S_{i}$ and their corresponding $\Pi^{rnd}$, $\Pi^{unc}$, and $\Pi^{max}$ over the set of rewired network community structures. We believe that $S_{i}^{unc}$ and the corresponding predictability $\Pi^{unc}$ are the most useful measures for structure uncertainty. The temporal-uncorrelated entropy considers the number of unique communities to which each node belongs in its community structures and its frequency of appearance in such communities. As shown in Figure 2, the $\Pi^{unc}$ increases as the corresponding Shannon entropy value decreases. The $S_{i}^{rnd}$ and $S_{i}$ are presented for completeness. It is important to note that the value of $S_{i}$, and its corresponding predictability $\Pi^{max}$, are calculated based on the assumption that the user will visit the most frequent community members first. Thus, these values may change under a different assumption for patterns of visitations.

### 5.3. Rewiring Using Fraction-Based and Frequency-Based Predicted Communities

To evaluate the quality of communities generated using fraction-based and frequency-based methods, we first find the rewiring step that results in the minimum Shannon entropy value. As shown for the Caviar and Sicilian networks in Figure 1, this value can be reached repeatedly as we continue rewiring, even when the entropy starts rising. For the experiments conducted in this section, we refer to the round of rewiring with the first iteration of rewiring at which the minimum Shannon entropy value is reached, as $i^{can}$. We proceed by applying the fraction-based and frequency-based community prediction methods on the set of rewired network community structures present in the rewiring step that directly precedes $i^{can}$. We will refer to the predicted communities of the fraction-based method as $C_{frac}$ and to the frequency-based method as $C_{freq}$. For all the experiments conducted using the frequency-based method, the user-defined parameter *Z*, defined in Section 3.3, is set to $\frac{R}{2}$.

We use the best candidate network $g^{can}$ in the set of rewired networks created by the rewiring step that directly precedes $i^{can}$. We find that using either the $C_{frac}$ or the $C_{freq}$ generates communities whose Shannon entropy is lower than communities generated by rewiring $g^{can}$ as shown in Figure 1. This demonstrates that the construction heuristic goes beyond the optimization achievable by the rewiring. We also find that $\Pi^{unc}$ and $\Pi^{max}$ increase for the networks rewired using $C_{frac}$ and $C_{freq}$. Table 2 shows that the usage of the $C_{frac}$-predicted communities results in the highest predictability for $\Pi^{unc}$ and $\Pi^{max}$ across the set of rewired network community structures. Accounting for both the frequency of occurrence between nodes in communities and the size of the communities in which the nodes occur together improves predictions of the community structure from the set of rewired networks.

### 5.4. Validation with LFR Benchmark Networks

While we have shown that the resulting $C_{frac}$- and $C_{freq}$-defined community structures have a lower Shannon entropy value and higher predictability, it is difficult to prove their superiority because of the absence of a ground-truth community structure with which to compare our communities $C_{frac}$ and $C_{freq}$. It is rarely possible to obtain a ground truth when working with covert networks due to their secretive nature. Hence, to validate our methods on networks with a ground-truth community structure, we generate LFR benchmark networks [26] that are statistically equivalent to either the Caviar or Sicilian networks but have a known ground-truth community structure.

The authors of [26] provide a tool to generate networks with a desired set of properties. These include the number of nodes *N*, the average degree *k*, the maximum degree maxk, the proportion of the total edge weight that is internal to communities muw, the proportion of total degree that is internal to communities mut, the minimum number of communities minc, the maximum number of communities maxc, and the average clustering coefficient *C*. We compute the values of these properties on the Caviar and Sicilian networks. For setting the values of minc and maxc, we run the Louvain [21] community detection algorithm 100 times on each network and find the maximum and minimum number of communities detected. Of those 100 Louvain community structures, we use the community structure that appears most frequently to compute muw and mut. The rest of the properties are computed directly from the Caviar and Sicilian networks. We enter these properties into the LFR benchmark tool to obtain a single network that is statistically equivalent to the Caviar network but has a ground-truth community structure defined by the tool. We repeat this process five times to create five separate LFR benchmark networks generated from the Caviar network. The same is performed for the Sicilian network. We will refer to these networks as the Caviar LFR networks and Sicilian LFR networks.

To provide a baseline result, on each of the ten LFR networks, we run the Louvain algorithm 100 times, collecting the community structures obtained in each run. Then, we compute the normalized mutual information (NMI) [27] score between each community structure produced by the Louvain algorithm and the ground-truth community structure of the LFR network. Next, we compute two measures over the obtained 100 NMI scores, the average and the median values as the baseline results. Finally, we run our repeated-rewiring construction heuristic on the LFR benchmark networks to obtain the $C_{frac}$- and $C_{freq}$-defined community structures with the lowest Shannon entropy and compute the NMI scores between these structures and the ground-truth community structures.

**Figure 3:**
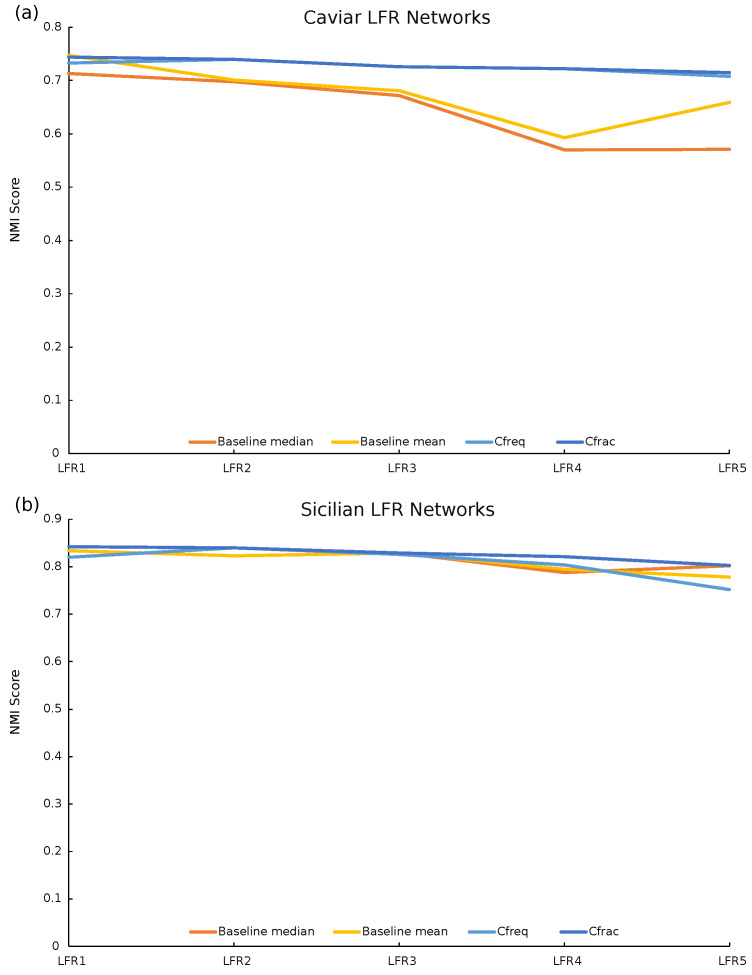
Five LFR benchmark networks are generated using the parameters of the Caviar (**a**) and Sicilian (**b**) networks. For each LFR network, we provide a baseline score by running Louvain community detection 100 times on the network and computing the normalized mutual information (NMI) scores of the detected communities versus the ground truth. The median and mean of these scores are plotted on the orange and yellow lines. Using our repeated-rewiring construction heuristic, we obtain the $C_{freq}$- and $C_{frac}$-defined community structures with the lowest Shannon entropy. The NMI scores for these structures compared to the ground truth are plotted on the light and dark blue lines. The $C_{frac}$-defined community structures have the highest NMI scores for nine out of the ten LFR networks. The only exception is the first Caviar-based LFR network where the $C_{frac}$ NMI score is 0.744 and the mean Louvain NMI score is 0.747, although this is a relatively tiny difference.

As shown in Table 3, the $C_{frac}$-defined community structures created by our repeated-rewiring construction heuristic have significantly improved the NMI scores on average compared to the baseline. As shown in Figure 3, the $C_{frac}$-defined community structures have the highest NMI score on 9 out of the 10 LFR networks. The only exception is the first Caviar-based LFR network where the $C_{frac}$ NMI score is 0.744 and the mean baseline NMI score is 0.747, but this is a relatively tiny difference. The $C_{frac}$ NMI score is still significantly higher than the median baseline NMI score of 0.713. Overall, these results demonstrate that the $C_{frac}$-defined community structures provided by our heuristic get closer to the ground truth than those provided by a traditional community detection algorithm.

The relative improvement in the NMI score is smaller on the Sicilian-based LFR networks than the Caviar networks. The Sicilian-based LFR network community structures also generally have higher NMI scores than those based on Caviar. This happens because the average degree of the Caviar network is 4.52 while for the Sicilian network it is 2.88. Low degrees of most nodes in the Sicilian network reduce the variance among the detected community structures.

## 6. Conclusions

The major contribution of this paper is the introduction of entropy measures of network community structure uncertainty and their use to establish limits of such uncertainties, defined as $\Pi^{rnd},\Pi^{unc},\Pi^{max}$ in Section 3.2. This enables us to search a pool of rewired networks for the one whose community structure has the lowest uncertainty, and even beyond this pool, when using our second heuristic, we assign each node to communities and network functions in the way that minimizes the expected cost of data uncertainty.

The abstract concept of network uncertainty is important, but even more important are the downstream consequences resulting from erroneous edge assignment. They can vary widely depending on which nodes are assigned to communities or functions that are different than predicted. In the case of criminal covert networks, such uncertainty may lead to the arrest of a low-level gang member instead of the leader. To address this challenge, we introduce a novel heuristic that constructs a community structure with the minimal expected cost of uncertainty of each node community membership and function. The cost function can be predefined or provided by the users based on the application. We showed three examples of such functions and developed a methodology that starts with noisy network data and maps nodes to communities and network functions that minimize the expected cost of data uncertainty.

In future work, we plan to extend the methodology to other domains in which networks are created from noisy data. We have already started to look at biomedical networks, in which data collections return massive volumes of noisy data, and the resilience of supply chains, in which diverse participants limit access to their proprietary data to preserve their competitive advantage.

## Figures and Tables

**Table 1:** The mean, range, and standard deviation of Shannon entropy are shown for all community structures found in networks rewired from the original Caviar, Jakarta, and Sicilian networks using the following values of $p_{B}=\left[0.5,0.75,0.875,0.9375\right]$. Each of the original networks was rewired 1000 times and divided into 10 groups with 100 networks each, to create 10 groups of results.

	$p_{B}$ Values	0.5	0.75	0.875	0.9375
Caviar	mean	3.640	3.246	2.727	2.169
	range	0.554	0.522	0.417	0.324
	σ	0.188	0.153	0.136	0.121
Jakarta	mean	2.043	1.379	0.944	0.621
	range	0.517	0.413	0.215	0.141
	σ	0.174	0.148	0.060	0.039
Sicilian	mean	6.908	6.908	6.907	6.826
	range	0	0	0.002	0.003
	σ	0	0	0.001	0.001

**Table 2:** We find that rewiring the network $g^{can}$ with the community structure $C^{can}$ reveals the community structures with the lowest observed Shannon entropy value. Using the BWRN generator [16], we further rewire $g^{can}$ using the $C_{frac}$- and $C_{freq}$-defined community structures in place of $C^{can}$. We find that the minimum Shannon entropy value of the set of rewired network community structures decreases in such cases. We also find that using the $C_{frac}$-defined community structure yields the set of rewired network community structures with the highest $\Pi^{unc}$ and the $\Pi^{max}$ predictability.

Community Structure	Shannon Entropy	$\Pi^{\mathrm{rnd}}$	$\Pi^{\mathrm{unc}}$	$\Pi^{\max}$
$C^{can}$	0.067	22.29%	46.95%	80.54%
$C_{frac}$	0.058	21.9%	50.49%	81.27%
$C_{freq}$	0.061	21.33%	52.39%	81.6%

**Table 3:** Five LFR benchmark networks are generated each based on the Caviar and Sicilian networks. As a baseline, the mean denoted for a vector $X_{l}$ as <$X_{l}$> and median denoted as $Median[X_{l}]$ and normalized mutual information (denoted as $NMI(CS_{1},CS_{2})$) scores are provided for a vector of 100 communities generated by Louvain community detection algorithm ($CS_{100}$), compared pairwise with the ground truth ($CS_{GT}$) on the Caviar and Sicilian-based LFR benchmark networks. Results for the heuristic-obtained Caviar and Sicilian-based LFR benchmark network structures with the lowest Shannon entropy are shown in the last two rows of the table. All four values are averaged over the 5 separate LFR benchmark networks. The $C_{frac}$-defined community structures with the lowest Shannon entropy have the highest average NMI scores.

Analysis of LFR Benchmark Ground-Truth Networks	Caviar	Sicilian
$<<NMI\left(CS_{100}\right)>/NMI\left(CS_{GT}\right)>$	0.676	0.812
$<<Median\left[NMI\left(CS_{100}\right)\right]>/NMI\left(CS_{GT}\right)>$	0.645	0.815
$NMI\left(C_{freq}\right)/NMI\left(CS_{GT}\right)$	0.726	0.808
$NMI\left(C_{frac}\right)/NMI\left(CS_{GT}\right)$	0.729	0.827

## Data Availability

The source code and data used in this study is openly available here: https://github.com/aamirusmandus/Network-Analytics-Enabled-by-Generating-a-Pool-of-Network-Variants-from-Noisy-Data, accessed on 22 July 2023.
